# Systematic review of diet quality scores including diet diversity in relation to major chronic diseases, obesity and mortality in healthy adults

**DOI:** 10.1007/s00394-026-03907-x

**Published:** 2026-04-28

**Authors:** Daniela V. Nickel, Franziska Jannasch, Matthias B. Schulze

**Affiliations:** 1 Department of Molecular Epidemiology, German Institute of Human Nutrition Potsdam-Rehbruecke, Nuthetal, Germany; 2 Institute of Nutritional Science, University of Potsdam, Potsdam, Germany

**Keywords:** Diet diversity, Diet quality, Major chronic diseases, Mortality, Obesity, High-income Western countries

## Abstract

**Purpose:**

Diet diversity is highlighted in dietary guidelines, yet diverse diets can vary substantially in quality. Understanding its role within overall diet quality is essential. This systematic review investigated diet quality scores incorporating a distinct diet diversity component (DQDS) and their associations with type 2 diabetes (T2D), cancer, cardiovascular diseases (CVD), obesity and all-cause mortality among healthy adults in high-income Western countries.

**Methods:**

A systematic search of the NCBI Database up to October 2025 identified prospective studies quantifying dietary intake and assessing a priori defined DQDS in relation to the outcomes of interest. Study quality was evaluated using the Scottish Intercollegiate Guidelines Network checklist. Due to substantial heterogeneity, findings were synthesised narratively.

**Results:**

Sixteen reports were included, applying eight DQDS, including the original Healthy Eating Index, Diet Quality Index variants, national Dietary Guidelines Indices and the Healthy Food Diversity-Index. Considerable variability existed in the diet quality dimensions measured (adequacy, balance, moderation, diversity) and in how diet diversity was operationalised (simple food-group counts or evenness-based metrics). Associations between DQDS and all-cause mortality (n = 5), cancer (n = 4), CVD (n = 3), obesity (n = 4) and T2D (n = 3) were inconsistent, with studies reporting null or inverse relationships.

**Conclusion:**

Current evidence remains insufficient to determine whether diet quality scores that include diet diversity are linked to chronic disease outcomes. Heterogeneity in the conceptualisation of both diet quality and diet diversity limits comparability across studies, highlighting the need for conceptually aligned, multidimensional DQDS in future research to clarify these relationships.

**Supplementary Information:**

The online version contains supplementary material available at 10.1007/s00394-026-03907-x.

## Introduction

Major chronic diseases - including cardiovascular diseases (CVDs), cancers and type 2 diabetes (T2D) - remain leading causes of death worldwide [1]. Obesity substantially increases the risk of these conditions [2], yet both are largely preventable through healthier lifestyles, particularly diet [3]. Accordingly, national and international policies promote healthy eating patterns that emphasise diet diversity to ensure a wide range of foods and adequate nutrient intake [3–9].

The Healthy Diets Monitoring Initiative (HDMI), jointly developed by the Food and Agriculture Organization of the United Nations (FAO), United Nations Children’s Fund (UNICEF) and World Health Organization (WHO), identified four core principles of a healthy diet: adequacy, balance, moderation and diversity [9, 10]. These principles highlight that diet diversity constitutes only one component of diet quality, alongside the need to ensure sufficient nutrient intake (adequacy), appropriate proportions of food groups (balance), and limitation of components associated with adverse health outcomes (moderation) [9, 10]. Similarly, Seligman et al. conceptualised diet quality as a multidimensional construct encompassing nutrient adequacy, nutrient density, macronutrient balance, diversity, moderation and safety [11], reinforcing that diversity forms only one dimension of overall diet quality.

Despite its recognised importance, the conceptualisation and measurement of diet diversity remain highly heterogeneous [12–14]. As outlined by Verger et al., existing indicators range from simple food or food-group counts to weighted or score-based metrics integrating intake frequency, quantity or healthfulness, and may differ in what they intend to measure versus what they actually reflect [12, 15]. Recent nutritional and ecological frameworks converge on three core dimensions of diet diversity: richness (the number of distinct foods or species consumed), evenness (the distribution of intake across foods or species) and dissimilarity/disparity (nutritional or functional dissimilarity between foods or species) [16–18]. These frameworks underscore that diet diversity is a multidimensional construct, and that the chosen metric critically shapes interpretation.

Because diets with greater diversity can vary substantially in overall quality, diversity measures alone may inadequately reflect diet quality [12, 14, 19, 20]. *A priori *diet quality scores that incorporate diversity alongside other dimensions (adequacy, balance and moderation) offer a more integrated assessment of diet quality. Such scores, including the original Healthy Eating Index (HEI, 1995) [21] and the Diet Quality Index – Revised (DQI-R) [22], contain an explicit, separate diversity component. In contrast, later HEI versions (e.g., HEI-2005) embed diversity within food group components reflecting updates to dietary guidelines [12, 23]. Because these versions no longer assess diet diversity as a distinct construct, they differ conceptually from scores that explicitly measure it. For this review, we therefore included only diet quality scores with a clearly defined, separate diversity component - here termed diet quality scores including diet diversity (DQDS).

To date, no systematic review has synthesised prospective evidence on *a priori *DQDS or examined how these indices measure and reflect the multidimensional nature of diet quality. This review therefore evaluates associations between higher versus lower DQDS and major health outcomes - cancer, CVD, mortality, obesity and T2D - in adults from high-income Western countries, and characterises how the identified DQDS measure key dimensions of diet quality.

## Methods

The protocol of the systematic review has been registered in PROSPERO: International prospective register of systematic reviews (registration number: CRD42022298739; https://www.crd.york.ac.uk/PROSPERO/). This systematic review is reported in accordance with the Preferred Reporting Items for Systematic Reviews and Meta-Analyses (PRISMA) guidelines [24].

### Data sources and search strategy

The NCBI (National Center for Biotechnology Information) Database was systematically searched for original reports until 14 October 2025 without restriction on publication date. The search strategy included the following full-text-keywords “Quantitative Index for Dietary Diversity”, “Dietary Diversity Score”, “Healthy Food Diversity”, “Food Variety Score”, “Dietary Variety Score”, “diet diversity”, “dietary diversity”, “food diversity”, “food variety”, “diet variety”, “dietary variety”, “Healthy Eating Index” and “Humans”. The following title-keywords were included to ensure exclusion of specific populations (“mice”, “mouse”, “rats”, “rat”, “animals”, “animal”, “murine”, “children”, “pregnant” or “pregnancy”). The full search strategy is detailed in Supplementary Statement 1. Search results were directly imported into EndNote (version 21). The reference lists of included reports and relevant reviews identified through the search strategy were screened to identify further reports.

### Eligibility criteria

The decision about including studies in the systematic review was based on the following eligibility criteria (Supplementary Table 1).

#### Population

Studies were included if they focused on the general adult population (≥ 18 years of age) from high-income Western countries (Supplementary Table 2). Studies involving children, adolescents, pregnant or breastfeeding women, patients with known diseases, or very specific populations (e.g., Indigenous people) were excluded.

#### Exposure and comparator

Eligible studies quantitatively assessed dietary intake using an *a priori* defined diet quality score that includes diet diversity (DQDS) as the exposure, with the comparison defined as higher DQDS versus lower DQDS.

#### Outcome

Studies investigating the following outcomes were eligible for inclusion: all-cause mortality, cancer, CVD, obesity, and T2D.

#### Study design

Only prospective studies were included. Other study designs were excluded.

Reports had to be published in either English or German language.

### Screening process

The relevance of records was assessed hierarchically as follows: First screening of titles, abstracts and full texts based on *a priori* defined inclusion and exclusion criteria, and broad data extraction was performed independently by two reviewers (DVN and student assistants). Disagreements were resolved through consensus discussions involving two other researchers (FJ and MBS). The primary reasons for excluding reports are listed in detail in Supplementary Table 3.

### Data extraction and synthesis

Data from eligible reports were extracted in a standardised approach developed by the authors and tabulated including the following information: first author; year of publication; title of the publication; study name, country, and design; age at baseline and sex of study population; exclusion and inclusion criteria; number of incident cases or deaths; analytic sample size; follow-up time; dietary assessment tool used; DQDS and definition; outcome measures and their definition; main statistical analysis methods; statistical parameter (risk ratio, odds ratio, hazard ratio, mean differences, 95 % confidence intervals (CIs) or standard errors, and p-values); adjustment variables. Data were synthesised narratively due to large heterogeneity in applied DQDS between the studies.

### Quality assessment of the included reports

The quality of eligible reports was assessed using the well-established Scottish Intercollegiate Guidelines Network (SIGN) checklist for cohort studies [25]. The checklist includes 14 questions covering various aspects of internal validity, with responses categorised as “Yes”, “No”, “Cannot Say”, or “Not Applicable”. “Not Applicable” (N.A.) was used when a question did not apply to the specific study design or outcome. For example, the item *“The likelihood that some eligible subjects might have the outcome at the time of enrolment is assessed and taken into account in the analysis”* was marked as “N.A.” when mortality was the outcome of interest, since this question is not relevant in that context. Such items were excluded from the overall quality rating. Reports were rated based on the following criteria: “High Quality” = reports with two or fewer questions answered with “No”; “Acceptable Quality” = reports with more than two questions answered with “No” but the majority of questions answered with “Yes”; “Low Quality” = all other reports, which were excluded from the systematic review. As part of the quality assessment, attention was given to whether studies appropriately adjusted for key confounders known to influence diet-disease associations [26, 27], including age, sex, socioeconomic status, smoking, alcohol consumption, physical activity, total energy intake, and body mass index (BMI). Where available, additional adjustments for metabolic or clinical factors such as blood pressure, blood lipids, or pre-existing disease were also considered important. Detailed information on the quality assessment of included reports is displayed in Supplementary Table 4.

## Results

### Study selection

**Fig. 1:**
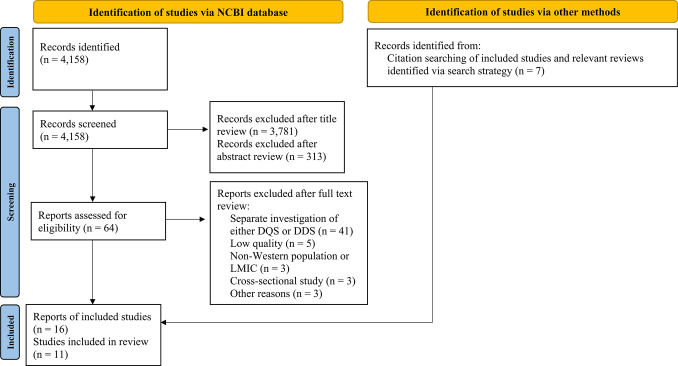
Flow diagram illustrating the literature search and study selection process (according to PRISMA 2020 [24]). The flow diagram was adapted from the PRISMA 2020 flow diagram [24]. DQS = Diet Quality Score; DDS = Diet Diversity Score; LMIC = low- or middle-income country; NCBI = National Center for Biotechnology Information

The search process is summarised in Fig. 1. The database search identified 4,158 records. Following the initial screening of titles, 377 abstracts were screened and 64 full texts were assessed, of which 14 reports initially met the inclusion criteria. Five reports were subsequently excluded due to insufficient data quality (Supplementary Table 5). Citation searching of included reports and relevant reviews identified seven additional eligible reports. In total, 16 reports comprising 11 unique prospective studies were included.

### Study characteristics

The included reports were published between 2000 and 2024 and comprised 423,496 participants with follow-up periods ranging from 2 to 23 years. Most studies were conducted in the USA [28–38], followed by Australia [39–41] and Europe [42, 43]. Sample sizes ranged from 285 to 71,058 participants.

Dietary intake was primarily assessed using semi-quantitative food frequency questionnaires (FFQ) [30, 32–35, 39–42], while others employed 24-hour dietary recalls (24hDR) [28, 29, 31, 43], quantitative diet history questionnaires [36, 37] or food records [38]. Reference periods for dietary assessments ranged from one day to one year. Baseline dietary assessments were conducted in all studies except one [30]. Seven studies collected repeated dietary data across two to five time points [30, 32, 34–38, 41], often summarising intake using cumulative averages or changes in DQDS over time.

The outcome definitions varied across studies. For example, CVD included myocardial infarction (MI) and/or stroke [34, 35, 42], while obesity outcomes ranged from change of body weight [38, 43] or waist circumference (WC) [38] to incidence of obesity (BMI ≥ 30 kg/m^2^) [43], body weight gain [36], general overweight (BMI ≥ 25 kg/m^2^), and abdominal obesity (WC ≥ 94 cm in men and WC ≥ 80 cm in women) [41].

Detailed study characteristics are provided in Table 1.

**Table 1:**
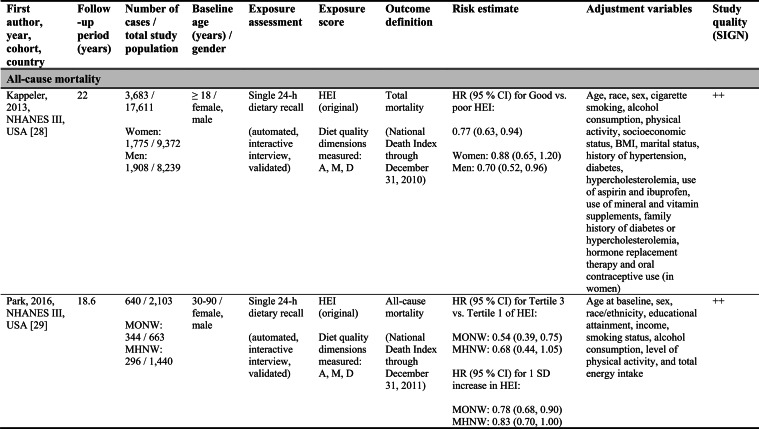

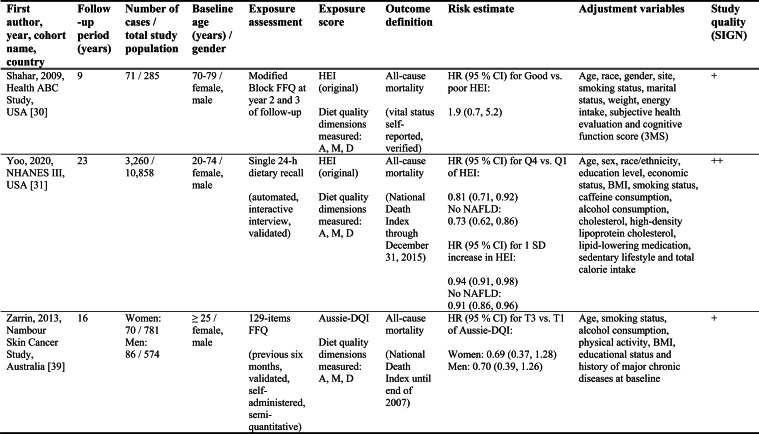

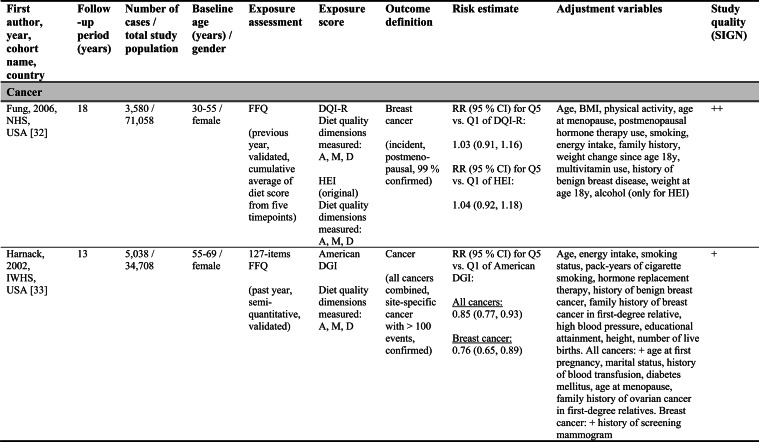

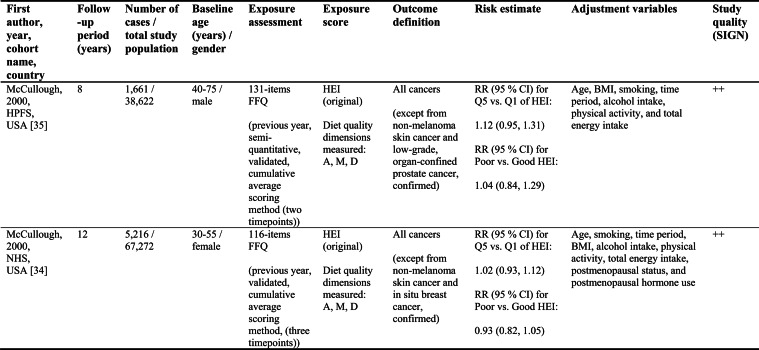

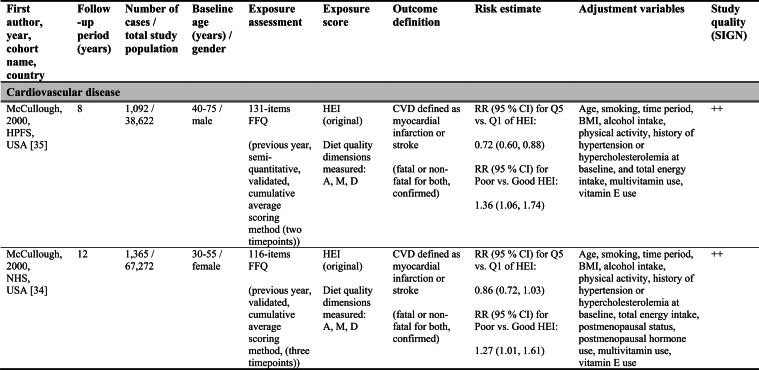

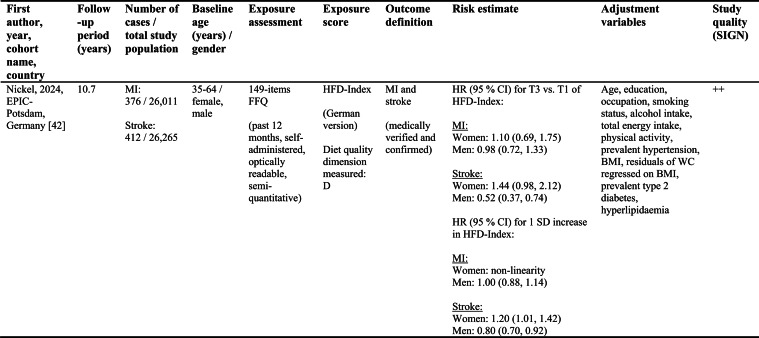

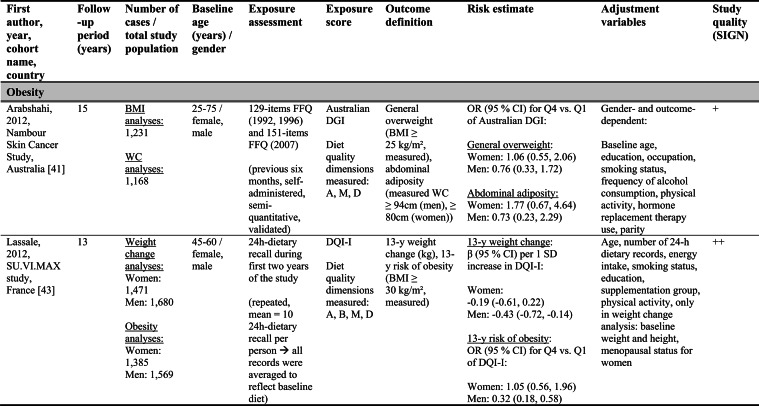

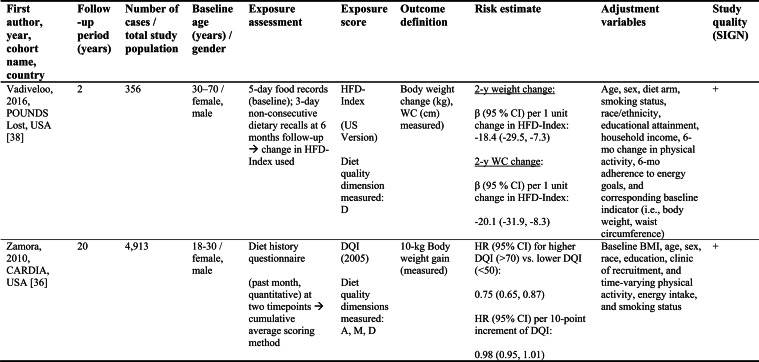

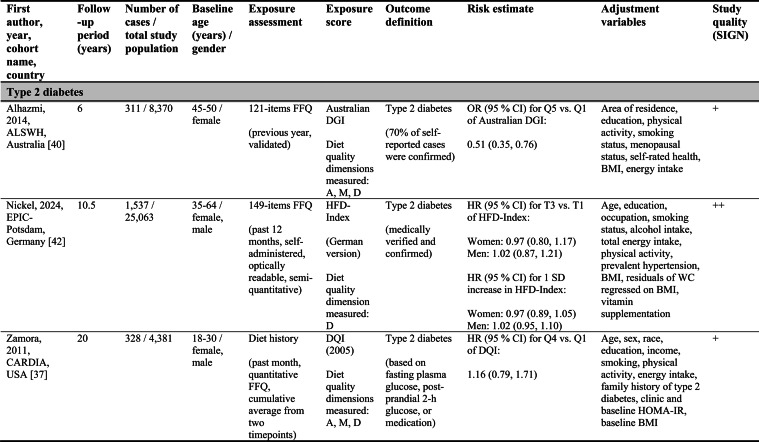
Summary characteristics of included reports investigating diet quality scores including diet diversity and chronic disease outcomes

A: Adequacy; ABC: Aging and Body Composition; ALSWH: Australian Longitudinal Study on Women's Health; B: Balance; American DGI: American Dietary Guidelines Index; Australian DGI: Australian Dietary Guideline Index; BMI: body mass index; CARDIA: Coronary Artery Risk Development in Young Adults; D: Diversity; DQI (-R,-I): Diet Quality Index (-Revised,-International); EPIC: European Prospective Investigation into Cancer and Nutrition; FFQ: food frequency questionnaire; HEI: Healthy Eating Index; HFD: Healthy Food Diversity-Index; HOMA-IR: Homeostatic Model Assessment for Insulin Resistance; HPFS: Health Professionals Follow-up Study; HR: hazard ratio; IWHS: Iowa Women's Health Study; M: Moderation; MHNW: metabolically healthy normal-weight; NAFLD: non-alcoholic fatty liver disease; NHANES: National Health and Nutrition Examination Survey; NHS: National Health Service; OR: odds ratio; RR: risk ratio; SD: standard deviation; SIGN: Scottish Intercollegiate Guidelines Network; SU.VI.MAX; SUpplémentation en VItamines et Minéraux AntioXydants; WC: waist circumference

## Summary of applied DQDS

Across the 16 included reports, eight distinct a priori defined DQDS were identified (Table 2 and 3). The original Healthy Eating Index (HEI, 1995) was the most frequently applied score (n = 7) [28–32, 34, 35], followed by the Australian Dietary Guideline Index (Australian DGI; n = 2) [40, 41], the Diet Quality Index (DQI; n = 2) [36, 37], the Healthy Food Diversity – Index (HFD-Index; n = 2) [38, 42], and single reports using the Australian DQI (Aussie-DQI; n = 1) [39], the American Dietary Guidelines Index (American DGI; n = 1) [33], the DQI-International (DQI-I; n = 1) [43] and the DQI-Revised (DQI-R; n = 1) [32].

**Table 2:**
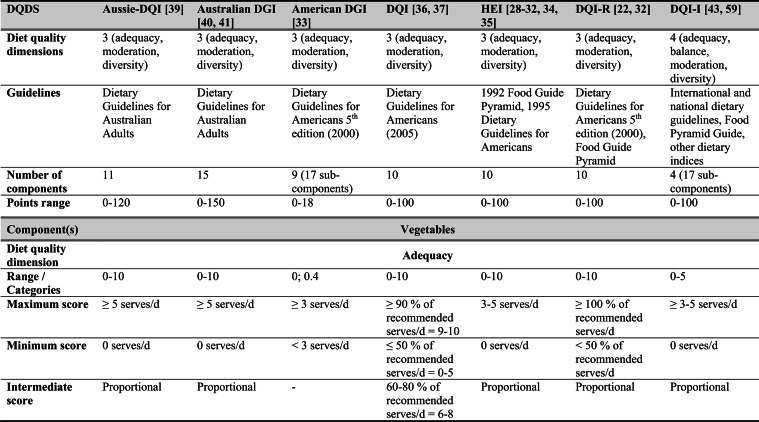

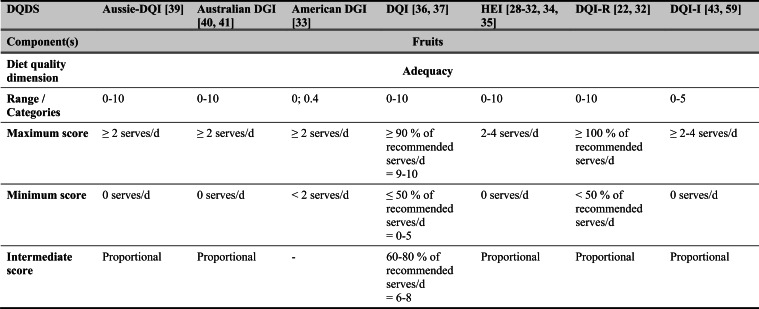

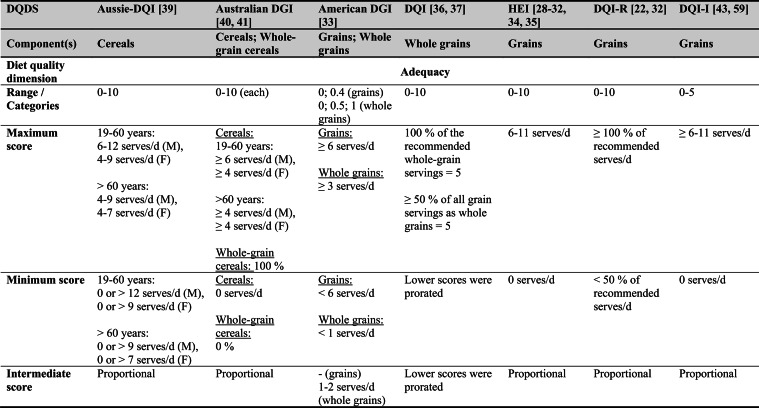

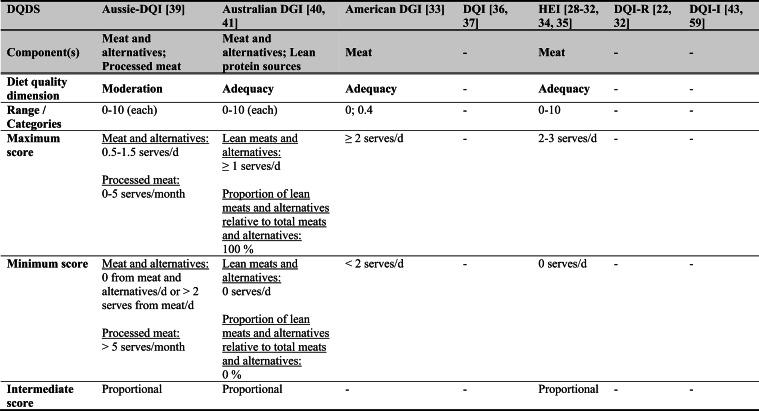

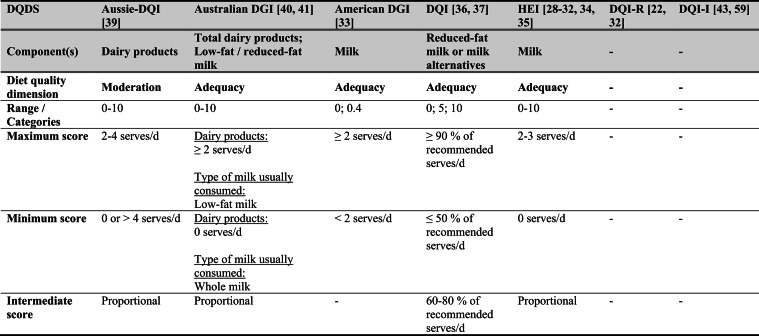

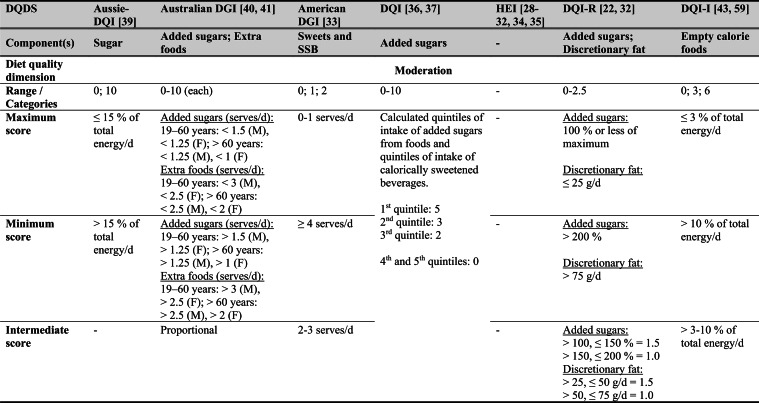

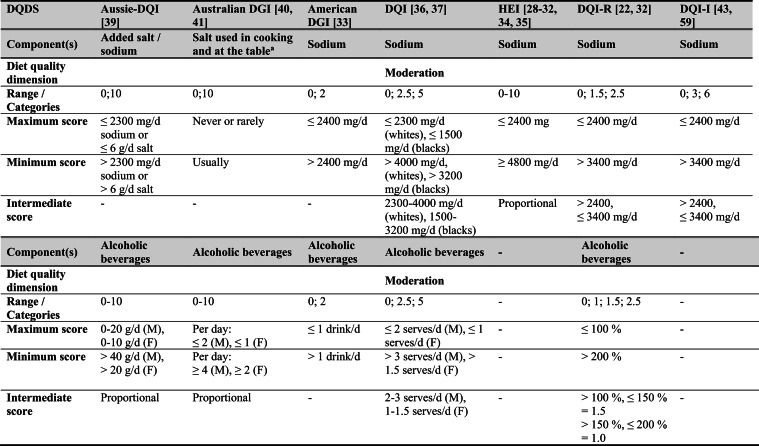

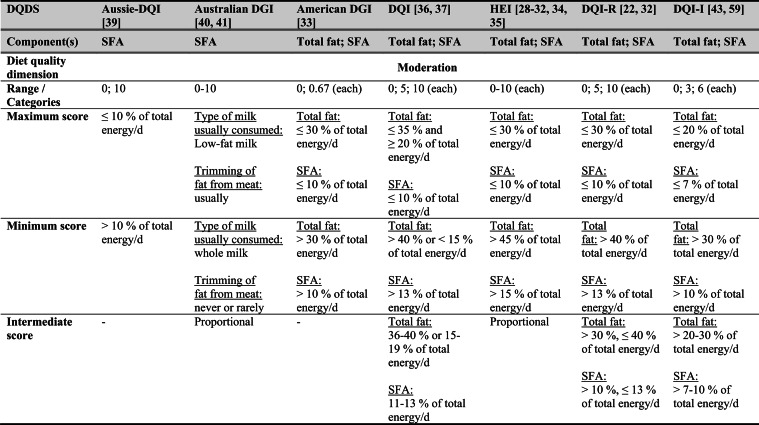

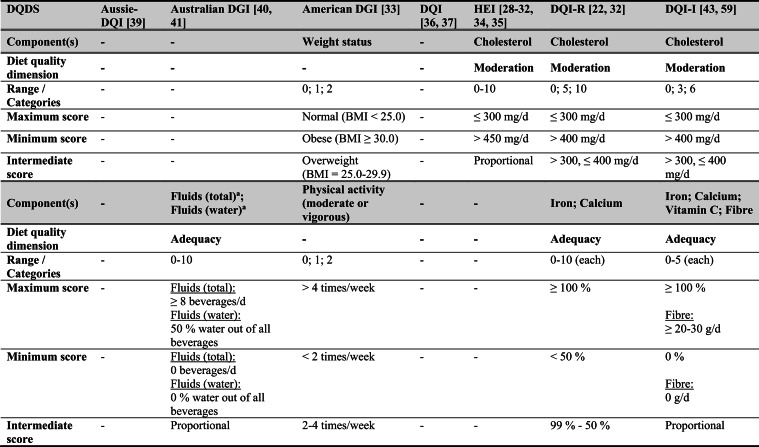

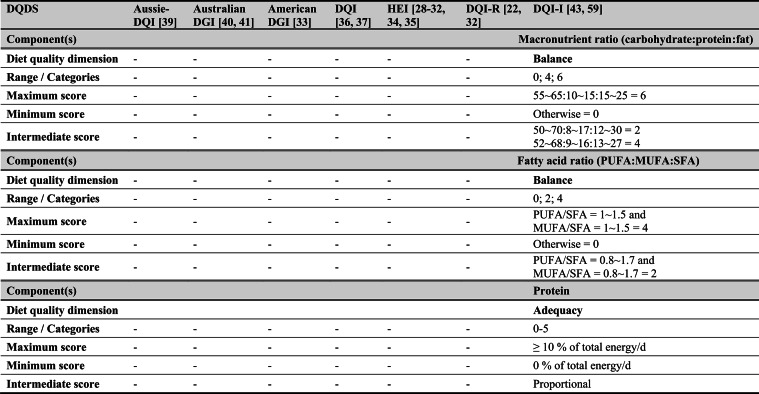

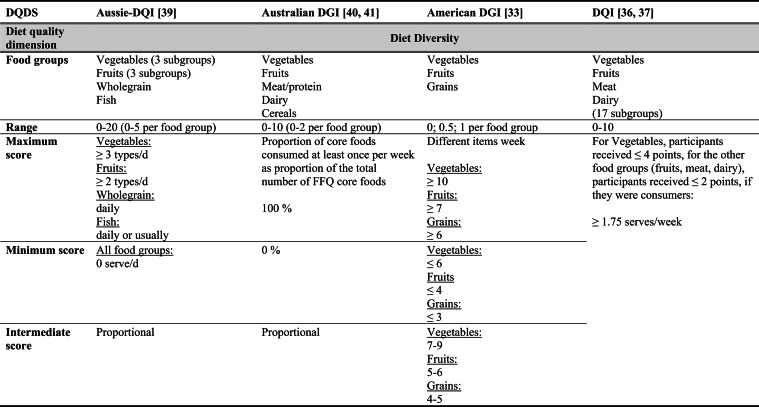

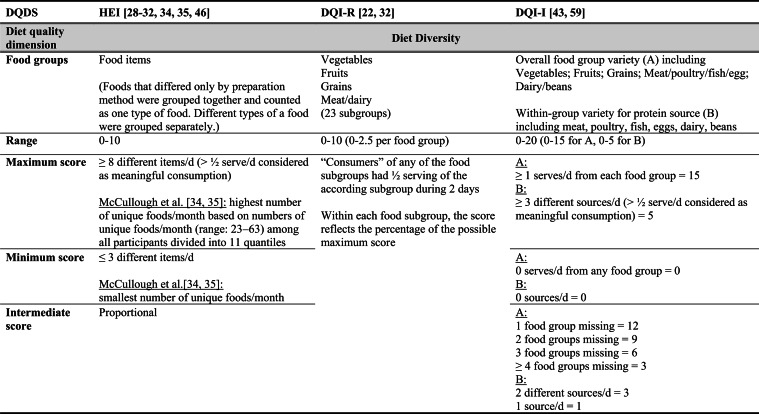
Definitions and scoring criteria of the identified component-based diet quality scores including diet diversity (DQDS)

^a^these components were excluded in the included reports

BMI = body mass index (in kg/m^2^); DGI: Dietary Guideline Index; DQI (-R,-I): Diet Quality Index (-Revised,-International); F: female; HEI: original Healthy Eating Index; HFD: Healthy Food Diversity-Index; M: male; MUFA = mono-unsaturated fatty acids; PUFA = poly-unsaturated fatty acids; SFA = saturated fatty acids; SSB = sugar-sweetened beverages.

The original HEI [28–32, 34, 35] based on the 1992 Food Guide Pyramid [44] and the 1995 Dietary Guidelines for Americans [45], consists of 10 components representing different aspects of a healthy diet [46]. The first five components evaluate alignment with Food Guide Pyramid servings for grains, vegetables, fruits, milk, and meat. Components 6 - 9 assess intake of total fat, saturated fat, cholesterol, and sodium. The tenth component measures dietary variety as the number of foods consumed in amounts equivalent to at least half a serving per day. A maximum score is assigned for consuming ≥ 8 different foods, a minimum score for ≤ 3 foods, and proportional scoring is applied for 3 - 8 foods [46]. Six other scores - Australian DGI [41], DQI [36, 37], DQI-I [43, 47], DQI-R [32], Aussie-DQI [39], and American DGI [33] - employed a similar approach, summing up multiple components reflecting dietary adequacy (e.g., fruit, vegetables, grains) or moderation (e.g., saturated fat, sodium) according to national dietary guidelines. The number of components ranged from 9 to 17 across scores. All included fruits, vegetables, and saturated fat, although additional components varied substantially. Some indices incorporated sodium, grains, dairy, sweets, alcohol, cholesterol, or total fat, while the DQI-I and DQI-R uniquely included specific micronutrients. Scoring criteria also differed: while many indices assigned proportional scores ranging from 0 to 10 points for meeting graded intake thresholds, others (e.g., the American DGI) used only two or three intake categories.

Regarding the diet diversity component, the majority of the identified DQDS summed up a varying number of food groups, ranging from 3 to 23, based on various cut-off criteria without considering relative amounts of different food groups within the overall diet [28–37, 39–41, 43]. In contrast, the HFD-Index was defined differently [19, 20]. Diet diversity was measured using the Berry-Index, which measures the distribution (evenness) of all food items in the diet. This was then multiplied by a health value, calculated as the sum of each food item's proportional contribution to total intake multiplied by a health factor based on either German [20, 42] or US dietary guidelines to reflect diet healthiness [19, 38]. As such, the HFD-Index uniquely integrates diversity, proportionality and healthiness into a single formula. Detailed scoring procedures for the US and German versions are presented in Table 3.

**Table 3:** Definitions and scoring criteria of the German and US Healthy Food Diversity – Index, which measure one diet quality dimension (diversity)

German Healthy Food Diversity – Index^a^ [20, 42]			US Healthy Food Diversity – Index^a^ [19, 38]		
Food group	Share of food subgroup, %	Health factors	Food group	Share of food subgroup, cup^b^	Health factors
Plant Foods (73 %)		0.73 x	Emphasises (78 %)		0.78 x
Vegetables / fruits / leaf salads / juices	36	0.36 = 0.2628	Whole grains	1.5/9.49 = 0.16	0.16 = 0.12
Wholemeal products / paddy	28	0.28 = 0.2044	Low-fat milk	3/9.49 = 0.32	0.32 = 0.25
Potatoes	20	0.20 = 0.1460	Vegetables	2.5/9.49 = 0.26	0.26 x
White-meal products / peeled rice	12	0.12 = 0.0876	Dark green vegetables	0.2/2.5 = 0.08	0.08 = 0.02
Snacks and sweets	4	0.04 = 0.0292	Red and orange vegetables	0.8/2.5 = 0.32	0.32 = 0.07
Animal foods (25 %)		0.25 x	Legumes	0.2/2.5 = 0.08	0.08 = 0.02
Fish / low-fat meat / low-fat meat products	36	0.36 = 0.090	Starchy vegetables	0.7/2.5 = 0.28	0.28 = 0.06
Low-fat milk / low-fat dairy products	28	0.28 = 0.070	Other vegetables	0.6/2.5 = 0.24	0.24 = 0.05
Milk / dairy products	20	0.20 = 0.050	Fruits	2/9.49 = 0.21	0.21 = 0.16
Meat products / sausages / eggs	12	0.12 = 0.030	Nuts, seeds and soya products	0.08/9.49 = 0.008	0.008 = 0.006
Bacon	4	0.04 = 0.010	Seafood	0.3/9.49 = 0.03	0.03 = 0.02
Fats and oils (2 %)		0.02 x	Oils	0.11/9.49 = 0.01	0.01 = 0.008
Oilseed rape / walnut oil	36	0.36 = 0.0072	Includes (20 %)		0.2 x
Wheat germ oil / soybean oil	28	0.28 = 0.0056	Meat	0.45/2.43 = 0.19	0.19 = 0.04
Corn oil / sunflower oil	20	0.20 = 0.0040	Poultry	0.38/2.43 = 0.16	0.16 = 0.03
Margarines / butter	12	0.12 = 0.0024	Eggs	0.1/2.43 = 0.04	0.04 = 0.01
Lard / vegetable fat	4	0.04 = 0.0008	Refined grains	1.5/2.43 = 0.62	0.62 = 0.12
			Limits (2 %)		0.02 x
			Discretionary solid fats	0.07/0.23 = 0.3	0.3 = 0.006
			Added sugar	0.16/0.23 = 0.7	0.7 = 0.01

HFD−Index =$$\left(1- \sum {s}_{i}^{2}\right)\cdot (\sum {hf}_{i} \cdot {s}_{i}$$)

${s}_{i}$= share of each individual food item$i$by weight of total food intake;${hf}_{i}$= health factor of each individual food item$i$

Berry-Index = $1- \sum {s}_{i}^{2}$; Health value = $\sum {hf}_{i} \cdot {s}_{i}$

^a^The health valuation of foods is the authors' interpretation of the national dietary guidelines (German Nutrition Society [20] or 2010 Dietary Guidelines for Americans [19]); ^b^based on specific intake recommendations in the 2000-kcal USDA Food pattern

Given these conceptual differences, the identified DQDS were grouped according to the diet quality dimensions they measure (adequacy, balance, moderation, diversity) [9]. This conceptual grouping revealed three distinct types of indices. The first group included DQDS that measure all four dimensions. Only the DQI-I met this criterion, as it measures adequacy, balance, moderation and diversity through distinct components. The second group included DQDS that measure adequacy, moderation and diversity but do not include a balance component. This category comprised the original HEI, the DQI, the DQI-R, the Australian DGI, the Aussie-DQI and the American DGI. The third group consisted of DQDS that primarily operationalised diversity. The HFD-Index (US and German versions) belonged to this category, as it primarily measured diversity. While the health value indirectly relates to some aspects of adequacy or moderation, the HFD-Index does not include explicit measures for other diet quality dimensions than diversity and are therefore considered primarily diversity-focused [15]. Detailed descriptions of all component-based DQDS are provided in Table 2 and the calculation of the HFD-Indices is presented separately in Table 3. The typology guided the synthesis of study results.

### Results of syntheses

**Fig. 2:**
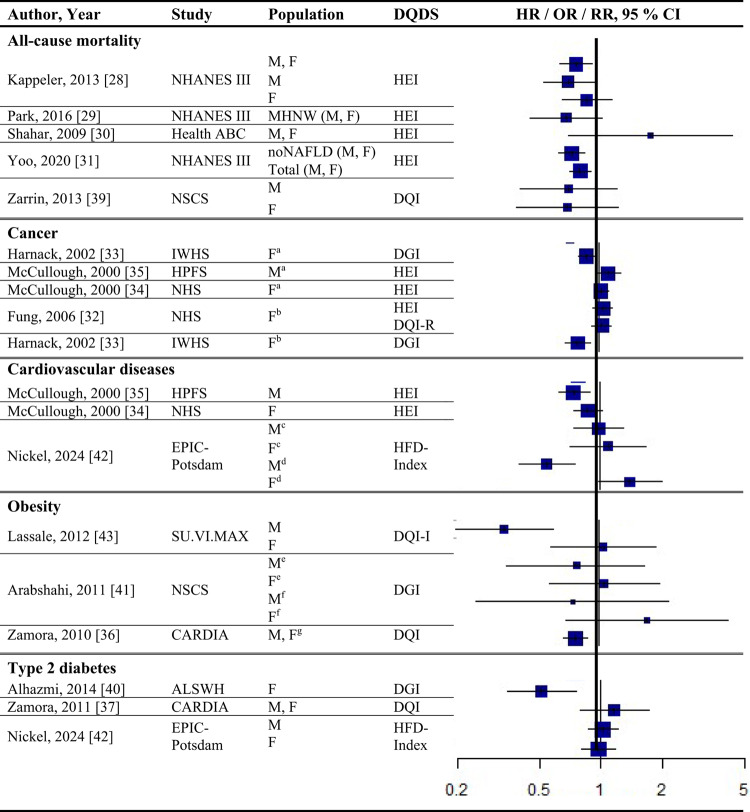
Forest plot displaying multiple-adjusted risk estimates as hazard ratio (HR), risk ratio (RR), or odds ratio (OR), and 95 % confidence interval (CI) of specified outcomes among participants with highest versus lowest diet quality score including diet diversity (DQDS). ABC: Aging and Body Composition; ALSWH: Australian Longitudinal Study on Women's Health; CARDIA: Coronary Artery Risk Development in Young Adults; DGI: Dietary Guideline Index; DQI (-R,-I): Diet Quality Index (-Revised,-International); EPIC: European Prospective Investigation into Cancer and Nutrition; F: female; HEI: original Healthy Eating Index; HFD: Healthy Food Diversity-Index; HPFS: Health Professionals Follow-up Study; IWHS: Iowa Women's Health Study; M: male; MHNW: metabolically healthy normal-weight; NAFLD: non-alcoholic fatty liver disease; NHANES: National Health and Nutrition Examination Survey; NHS: National Health Service; NSCS: Nambour Skin Cancer Study; SU.VI.MAX; SUpplémentation en VItamines et Minéraux AntioXydants. ^a^all cancers; ^b^breast cancer; ^c^myocardial infarction; ^d^stroke; ^e^general overweight; ^f^abdominal adiposity; ^g^10-kg body weight gain

Study quality ranged from acceptable to high (Supplementary Table 4). Detailed findings are presented in Table 1 and illustrated in Fig. 2.

#### DQDS measuring adequacy, balance, moderation and diversity

Only one study applied the DQI-I [43]. Conducted in France, this study examined body weight change and obesity risk over 13 years. Higher DQI-I scores were associated with lower weight gain and reduced obesity risk among men, whereas no associations were observed among women. This study was rated as high quality. No studies using the DQI-I investigated all-cause mortality, cancer, CVD or T2D.

#### DQDS measuring adequacy, moderation and diversity

This was the largest typology group of DQDS and included studies covering all five outcome categories. The overall quality of the studies ranged from acceptable to high.

#### All-cause mortality

Five reports investigated all-cause mortality. Three high-quality US reports based on the NHANES III population identified inverse associations between higher original HEI scores and all-cause mortality risk in the overall study population [28, 31], in men [28, 29], and in metabolically obese and normal-weight participants [29]. In these reports, the reduced risk of all-cause mortality ranged from 19 to 23 % when comparing extreme quantiles of original HEI [28, 29, 31]. One other US cohort using the original HEI [30] and one Australian study using the Aussie-DQI [39] reported no clear associations. Both were of acceptable quality.

#### Cancer

Four reports investigated cancer outcomes in three individual US study populations (two female and one male population) [32–35]. Three high-quality studies applying the original HEI [32, 34, 35] or DQI-R [32] found no associations with breast or all cancers. One acceptable-quality study using the American DGI reported inverse associations with all cancers and breast cancer in women [33].

#### Cardiovascular diseases

Two high-quality US studies using the original HEI [34, 35] identified inverse associations with CVD incidence, which was more pronounced in men (28 % reduced risk) than women [34, 35]. The comparison of *a priori* defined original HEI categories showed similar patterns.

#### Obesity-related outcomes

Two acceptable-quality studies investigated obesity outcomes [36, 41]. One US study identified inverse associations between DQI and 10 kg body weight gain over 20 years [36]. One Australian study using the Australian DGI found no clear associations with overweight or abdominal adiposity [41].

#### Type 2 diabetes

Two acceptable-quality studies investigated T2D incidence [37, 40]. Australian women with higher Australian DGI scores had a 49 % lower T2D risk when comparing extreme quintiles [40]. A US study using the DQI reported no association with T2D [37].

#### DQDS measuring diversity

##### Cardiovascular diseases

The high-quality German study found inverse associations with stroke in men but no associations with MI. In women, higher HFD-Index scores were associated with increased stroke risk [42].

##### Obesity-related outcomes

The high-quality US HFD-Index study reported inverse associations with 2-years changes in body weight and waist circumference [38]. The German HFD-Index study did not investigate obesity.

##### Type 2 diabetes

The German HFD-Index showed no association with T2D incidence [42].

##### All-cause mortality and cancer

No HFD-Index studies investigated these outcomes.

Overall, associations differed within the two typology groups for which multiple studies were available. No broader conclusions can be drawn for the four-dimension DQDS because only a single DQI-I study was identified.

## Discussion

To our knowledge, this is the first systematic review to synthesise prospective evidence on *a priori* diet quality scores that include an explicit component of diet diversity (DQDS) and their associations with major chronic diseases, obesity and mortality in adults from high-income Western countries. Across 16 reports comprising 11 individual studies, we found no consistent evidence that higher DQDS were associated with lower risks of all-cause mortality, cancer, CVD, obesity or T2D. Although several reports identified inverse associations, findings varied substantially across outcomes, populations and score types. Much of this inconsistency appears to stem from substantial methodological heterogeneity in how diet quality and diet diversity were defined, operationalised and measured.

Our findings differed from earlier meta-analyses that investigated either diet quality or diet diversity in isolation [14, 48]. For all-cause mortality, Mozaffari et al. reported inverse associations with both total diet diversity (RR: 0.78; 95 % CI: 0.64, 0.96; I^2^ = 97 %) and healthy food diversity (RR: 0.84; 95 % CI: 0.73, 0.96; I^2^ = 61 %) [14]. Diet quality has also been associated with lower mortality risk (RR: 0.80; 95 % CI: 0.79, 0.82; I^2^ = 68 %) [48]. However, when analyses were restricted to the original HEI - the HEI version containing a separate diversity component - no association with all-cause mortality was found (RR: 1.05; 95 % CI: 0.45, 2.45; I^2^ = 67 %) [48], consistent with the mixed findings in our review.

For cancer, earlier reviews similarly found no pooled association for diet diversity [14], whereas higher diet quality was associated with lower cancer risk (RR: 0.87; 95 % CI: 0.84, 0.91; I^2^ = 76 %) [48]. Again, subgroup analyses restricted to the original HEI showed no association (RR: 1.02; 95 % CI: 0.89, 1.17; I^2^ = 46 %) consistent with our included studies [48].

For CVD, meta-analyses reported modest inverse associations with diversity (RR: 0.93; 95 % CI: 0.86, 1.00; I^2^ = 32 %) [14] and stronger associations with diet quality (RR: 0.81; 95 % CI: 0.78, 0.85; I^2^ = 42 %) [48]. A pooled analysis investigating only the original HEI and CVD risk (incidence or mortality) reported an RR of 0.80 (95 % CI: 0.71, 0.90, I^2^ = 0 %) [28, 34, 35, 48]. Although we included the same studies [34, 35], we investigated incidence and mortality separately, yielding different results. Variation in study populations, some including only men, others only women, may also contribute to inconsistent findings.

Evidence for obesity has been inconsistent. Similar to our findings, previous reviews identified inconsistent associations across heterogenous studies [13, 49–51]. Diet diversity was often positively associated with overweight or obesity, leading previous reviews to emphasise the need to distinguish between diversity in healthy foods and unhealthy foods [13, 50, 51].

For T2D, an earlier systematic review described inconclusive associations for overall diet diversity, but suggested potential benefits of diversity within specific food groups, particularly fruits and vegetables [13]. Diet quality demonstrated a clear relationship with lower T2D risk (RR: 0.81; 95 % CI: 0.78, 0.85; I^2^ = 76 %) [48].

Overall, comparisons with previous reviews are challenging because earlier syntheses did not examine combined measures of diet quality and diversity. They assessed these constructs separately and therefore included many studies that did not consider their interplay. Moreover, earlier meta-analyses pooled studies with considerable variation in dietary assessment tools, scoring methods, outcome definitions and population characteristics - limitations that affected our ability to compare findings.

A major reason for inconsistent findings in our review was the substantial heterogeneity in dietary assessment. Studies applied different dietary assessment tools and reference periods, ranging from single-day 24hDRs to year-long FFQs. FFQs may underestimate habitual diet diversity because of finite food lists, whereas single 24hDRs may not accurately reflect habitual diet diversity given day-to-day variation [52]. Nearly half of the studies relied only on baseline dietary assessment, which increases misclassification risk if diets change over time [53]. Combining multiple assessment methods and collecting repeated dietary data across follow-up would better capture both diet quality and diversity.

Beyond dietary assessment, the eight DQDS varied substantially in their conceptual structure. Based on recognised frameworks describing adequacy, balance, moderation and diversity as core principles of healthy diets [9, 10], three typological groups emerged: (1) scores measuring all four dimensions (DQI-I), (2) scores measuring adequacy, moderation and diversity but not balance (HEI, DQI, DQI-R, American DGI, Australian DGI, Aussie-DQI), and (3) scores measuring only diversity (HFD-Index). These dimensional differences influence interpretation. Scores measuring multiple diet quality dimensions may yield different associations than diversity-focused measures, whereas scores in which diversity contributes minimally to total points may allow diets with low diversity but high adequacy or moderation to achieve moderate overall scores. Conversely, the HFD-Index, although explicitly diversity-focused, incorporates proportionality and a guideline-based health value indirectly reflecting aspects of adequacy and moderation despite lacking explicit components for these dimensions. Such variability in what DQDS measure versus what they reflect [15] helps to explain the heterogeneous associations across typology groups. Additional variability across DQDS stemmed from differences in underlying dietary guidelines (various editions of the US, Australian or German guidelines), the number and type of components and their scoring criteria. Most DQDS were constructed by summing several components reflecting food groups, macro- or micronutrients, other nutrients, or lifestyle factors, together with a diversity component. Component scoring ranged from proportional scales (0 - 10 points) to simple categorical classifications, producing differing score ranges and distributions.

A further major source of variation was the operationalisation of diet diversity. Most DQDS counted the number of food groups consumed above predefined thresholds or simple food counts, which broadly captures only richness [28–33, 36, 37, 39–41, 43]. In contrast, the HFD-Index used the Berry-Index which measures the evenness of intake distribution across all consumed foods [18, 20, 38, 42]. As previously investigated, a potentially more precise measure of richness, the Dietary Species Richness was inversely associated with all-cause and cause-specific mortality even after adjustment for established diet quality components in a large European cohort [54]. This suggests that the inconsistent associations in our review may partly reflect limitations in how richness is measured within DQDS, rather than a lack of true association. Overall, these differences highlight the need for future studies to use diet quality scores with clearer and more consistent coverage of adequacy, balance, moderation and diversity, and to operationalise diet diversity as a distinct, multidimensional construct that is appropriately weighted within composite scores.

An additional contribution of this review is to highlight how the conceptualisation of diet diversity within diet quality scores has evolved. As noted by Verger et al. [12], food-based dietary guidelines have shifted over time from addressing adequacy, balance, moderation and diversity as distinct principles to embedding all four dimensions within each food-group recommendation. This shift is reflected in the HEI: the original HEI (1995) included an explicit diversity component, whereas later versions (HEI-2005 onwards) no longer treat diversity as a separate component but incorporate variety within food-group components deemed most beneficial according to updated guidelines (e.g., subtypes of vegetables or whole versus refined grains) [12, 55]. These changes complicate comparability across HEI versions and justified our decision to focus on diet quality scores with a clearly separated diversity component.

Despite inconsistent findings, several mechanisms may explain why a diverse, high-quality diet could support better health. Greater variety within healthy food groups can increase the intake of bioactive compounds such as dietary fibre, phytochemicals and antioxidants, which may influence cardiometabolic pathways including inflammation, lipid metabolism and blood pressure regulation [42, 56, 57]. A diverse range of nutrient-dense foods may also enhance satiety and support adherence to healthy eating patterns [38, 50, 56, 58]. These potential mechanisms warrant further investigation to clarify the pathways through which diet diversity embedded within high-quality diets may affect chronic disease risk.

This review has several limitations. Despite systematic screening, some relevant studies may have been missed if diet diversity was embedded within broader diet quality scores but not explicitly described. The search was limited to a single electronic database. Although this database provides broad coverage of major nutrition and epidemiology journals, and reference lists of included reports and relevant reviews were systematically screened to minimise the likelihood of missing additional studies, searching additional databases might have identified further records. Relatedly, the search applied only the MeSH term “Humans” to exclude animal studies. Incorporating additional MeSH exclusions may have further refined retrieval. Reports published in languages other than English or German were not included. While two reviewers were involved in the literature search, screening, and broad data extraction, a single reviewer conducted detailed data extraction and quality assessment of studies. Although this process followed a rigorous methodology, it could introduce potential bias or inconsistencies. Discrepancies were resolved through consensus discussions with additional reviewers. A pooled analysis was not conducted due to substantial heterogeneity in exposure assessment and outcome definitions among the included studies. The limited number of reports and the potential for sample overlap, such as the reuse of NHANES III data on mortality, hindered stratified subgroup analyses.

The main strength of this review is that it is the first comprehensive examination focusing specifically on diet quality scores, including diet diversity, and their associations with health outcomes. Only prospective studies were included, as they can establish temporal sequences and provide longitudinal insights, thereby strengthening the review's ability to draw causal conclusions on associations with health outcomes. Each included report underwent rigorous quality assessment using an established checklist, ensuring that only acceptable-to-high-quality evidence was considered. The review did not impose any time restrictions on the included studies, allowing for a comprehensive analysis of both historical and contemporary research. By developing and applying a typology based on adequacy, balance, moderation and diversity, we provided conceptual clarity on how the identified DQDS differ and where measurement gaps persist.

Overall, current evidence is insufficient to draw definitive conclusions about the combined role of diet quality and diet diversity in major health outcomes. Differences in dietary assessment and in how scores measured key diet quality dimensions, including diversity, limit comparability across studies. Future research should use conceptually aligned scores, operationalise diet diversity as a distinct and appropriately weighted dimension, apply repeated and comprehensive dietary assessments and include diverse populations to strengthen future dietary recommendations.

## Supplementary Information

Below is the link to the electronic supplementary material.

Supplementary Material 1

## Data Availability

The data supporting the findings of this study are available within the article and its supplementary material.

## Abbreviations

24hDR: 24-hour dietary recall
AHEI: Alternate Healthy Eating Index
Aussie-DQI: Australian Dietary Quality Index
BMI: Body mass index
CI: Confidence interval
CVD: Cardiovascular diseases
DASH: Dietary Approaches to Stop Hypertension
DDS: Diet Diversity Score
DGI: Dietary Guideline Index
DQDS: Diet quality score including diet diversity
DQI: Diet Quality Index
DQI-I: Diet Quality Index - International
DQI-R: Diet Quality Index - Revised
DQS: Diet Quality Score
FAO: Food and Agriculture Organization
FFQ: Food frequency questionnaire
GRADE: Grading of Recommendations Assessment, Development and Evaluation
HDMI: Healthy Diets Monitoring Initiative
HEI: Healthy Eating Index
HFD-Index: Healthy Food Diversity - Index
LMIC: Low- or middle-income country
MI: Myocardial infarction
NCBI: National Center for Biotechnology Information
NHANES: National Health and Nutrition Examination Survey
SIGN: Scottish Intercollegiate Guidelines Network
T2D: Type 2 diabetes
UNICEF: United Nations Children’s Fund
USDA: U.S. Department of Agriculture
WC: Waist circumference
WHO: World Health Organization
