# Supplementary material for: Systematic review of diet quality scores including diet diversity in relation to major chronic diseases, obesity and mortality in healthy adults
Source: Eur J Nutr. 2026 Apr 28;65(4):125. doi: 10.1007/s00394-026-03907-x (PMC13124849; doi:10.1007/s00394-026-03907-x)
Supplement: Supplementary file 1 — Supplementary Material 1 [file 394_2026_3907_MOESM1_ESM.docx]

Systematic review of diet quality scores including diet diversity in relation to major chronic diseases, obesity and mortality in healthy adults

European Journal of Nutrition

Daniela V Nickel^1,2*^, Franziska Jannasch^1^, Matthias B Schulze^1,2^

^1^Department of Molecular Epidemiology, German Institute of Human Nutrition Potsdam-Rehbruecke, Nuthetal, Germany

^2^Institute of Nutritional Science, University of Potsdam, Potsdam, Germany

*****Correspondence: Daniela V Nickel, Daniela.Nickel@dife.de

**Supplementary material**

Table of Contents

[Supplementary Statement 1 Search syntax 2](#_Toc215582478)

[Supplementary Table 1 PECOS criteria for eligibility of studies 2](#_Toc215582479)

[Supplementary Table 2 High-income Western countries eligible for inclusion 3](#_Toc215582480)

[Supplementary Table 3 List of primary reasons for exclusion of studies 4](#_Toc215582481)

[Supplementary Table 4 Quality assessment of included reports by the Scottish Intercollegiate Guidelines Network (SIGN) checklist 5](#_Toc215582482)

[Supplementary Table 5 Quality assessment of reports by the Scottish Intercollegiate Guidelines Network (SIGN) checklist which were excluded due to low quality 8](#_Toc215582483)

[References 10](#_Toc215582484)

## Supplementary Statement 1 Search syntax

(((((((((((("Quantitative Index for Dietary Diversity") OR "Dietary Diversity Score") OR "Healthy Food Diversity") OR "Food Variety Score") OR "Dietary Variety Score") OR "diet diversity") OR "dietary diversity") OR "food diversity") OR "food variety") OR "diet variety") OR "dietary variety") OR "Healthy Eating Index" AND (Humans[Mesh])) NOT ((((((((((mice[Title]) OR mouse[Title]) OR rats[Title]) OR rat[Title]) OR animals[Title]) OR animal[Title]) OR murine[Title]) OR children[Title]) OR pregnant[Title]) OR pregnancy[Title] AND (Humans[Mesh]))

## Supplementary Table 1 PECOS criteria for eligibility of studies

|  | **Inclusion criteria** | **Exclusion criteria** |
| --- | --- | --- |
| **Population** | General healthy human population  ≥ 18 years of age  High-income Western countries (Supplementary Table 2) | Children and adolescents  Pregnant or breastfeeding women  Patients with known diseases  Very specific populations (e.g. Indigenous people)  Low- and middle-income non-Western countries  Animal studies |
| **Exposure** | A priori defined diet quality score including diet diversity (DQDS; diet diversity reflected by ≥ three different food groups) based on quantitatively assessed dietary intake | A priori defined diet quality score without diet diversity or vice versa  A posteriori defined diet quality and/or diet diversity  Dietary intake not assessed quantitatively  DQDS with diet diversity reflecting less than three food groups |
| **Comparison** | High DQDS versus low DQDS | No comparison |
| **Outcome** | All-cause mortality, cancer, cardiovascular disease, myocardial infarction, stroke, obesity, and type 2 diabetes | Biomarkers of cardiometabolic risk |
| **Study design** | Prospective observational studies | Cross-sectional and retrospective case-control studies |

## Supplementary Table 2 High-income Western countries eligible for inclusion

| Andorra | Germany | Panama |
| --- | --- | --- |
| Antigua and Barbuda | Gibraltar | Poland |
| Australia | Greece | Portugal |
| Austria | Hungary | San Marino |
| Bahamas | Iceland | Slovac Republic |
| Barbados | Ireland | Slovenia |
| Belgium | Italy | Spain |
| Canada | Latvia | St. Kitts and Nevis |
| Chile | Liechtenstein | Sweden |
| Croatia | Lithuania | Switzerland |
| Cyprus | Luxembourg | Trinidad and Tobago |
| Czech Republic | Malta | United Kingdom |
| Denmark | Monaco | United States |
| Estonia | Netherlands | Uruguay |
| Finland | New Zealand |  |
| France | Norway |  |

This list was constructed in 2019 according to the World Bank List of Economies [1] and the “Rich West” Western countries as defined by the World Population Review [2]

## Supplementary Table 3 List of primary reasons for exclusion of studies (full-text level)

| **Reasons for exclusion** | **Number of studies** |
| --- | --- |
| Case-control study design | 1 [3] |
| Cross-sectional study design | 3 [4-6] |
| Separate investigation of either diet quality or diet diversity | 39 [7-47] |
| Diet diversity component only reflecting less than three food groups | 1 [48] |
| Reviews | 1 [49] |
| Non-western or low-/middle income country | 3 [41, 50-52] |
| Low quality, potential high risk of bias | 5 [53-57] |

## Supplementary Table 4 Quality assessment of included reports by the Scottish Intercollegiate Guidelines Network (SIGN) checklist

| **First author, year, study name** | **Alhazmi, 2014**  **ALSWH [58]** | **Arabshahi, 2011 Nambour Skin Cancer Study [59]** | **Fung, 2006**  **NHS [60]** | **Harnack, 2002**  **IWHS [61]** | **Kappeler, 2013**  **NHANES III [62]** |
| --- | --- | --- | --- | --- | --- |
| Appropriate, clearly focused question | Y | Y | Y | Y | Y |
| Selection from source populations comparable in all respects other than the factor under investigation | Y | Y | Y | Y | Y |
| Indication for how many of the people asked to take part did so | Y | Y | N | Y | Y |
| Likelihood of the outcome at the time of enrolment is assessed and considered | C.S. | N.A. | Y | Y | N.A. |
| Percentage of individuals that dropped out before study completion | - | - | < 5 % | 21 % | N.A.^b^ |
| Comparison between full participants and those lost to follow-up, by exposure status | C.S. | Y | N | N | N.A. |
| Clearly defined outcome | Y | Y | Y | Y | Y |
| Assessment of outcome blind to exposure status | N.A. | N.A. | N.A. | N.A. | N.A. |
| When blinding not possible, recognition that knowledge of exposure status could have influenced assessment of outcome | Y | Y | Y | Y | Y |
| Reliable exposure assessment | Y | C.S. | Y | Y | Y |
| Evidence from other sources, that method of outcome is valid and reliable | Y | N.A. | Y | N.A. | N.A. |
| Exposure level assessed more than once | N | Y | Y | N | N |
| Main confounders identified and considered | C.S. | C.S. | Y | Y | Y |
| Confidence intervals provided | Y | Y | Y | Y | Y |
| **Quality assessment** | **+** | **+** | **++** | **+^a^** | **++** |

**Supplementary Table 4** Continued

| **First author, year, study name** | **Lassale, 2012, SU.VI.MAX study [63]** | **McCullough, 2000**  **NHS [64]** | **McCullough, 2000**  **HPFS [65]** | **Nickel,**  **2023,**  **EPIC-Potsdam [66]** | **Park, 2016**  **NHANES III [67]** | **Shahar, 2009**  **Health ABC [68]** |
| --- | --- | --- | --- | --- | --- | --- |
| Appropriate, clearly focused question | Y | Y | Y | Y | Y | Y |
| Selection from source populations comparable in all respects other than the factor under investigation | C.S. | Y | Y | Y | Y | Y |
| Indication for how many of the people asked to take part did so | Y | N | N | Y | Y | N |
| Likelihood of the outcome at the time of enrolment assessed & considered | Y | Y | Y | Y | N.A. | N.A. |
| Percentage of individuals that dropped out before study completion | 11.6 % | <5 % | 3 % | < 10 % | N.A.^b^ | N.A.^b^ |
| Comparison between full participants and those lost to follow-up, by exposure status | N | N | N | N | N.A. | N |
| Clearly defined outcome | Y | Y | Y | Y | Y | Y |
| Assessment of outcome blind to exposure status | N.A. | Y | Y | N.A. | N.A. | N.A. |
| When blinding not possible, recognition that knowledge of exposure status could have influenced assessment of outcome | Y | Y | Y | Y | Y | Y |
| Reliable exposure assessment | Y | Y | Y | Y | Y | C.S. |
| Evidence from other sources, that method of outcome is valid & reliable | N.A. | Y | Y | Y | N.A. | N.A. |
| Exposure level is assessed more than once | Y | Y | Y | N | N | Y |
| Main confounders identified and considered | Y | Y | Y | Y | Y | Y |
| Confidence intervals provided | Y | Y | Y | Y | Y | Y |
| **Quality assessment** | **++** | **++** | **++** | **++** | **++** | **+** |

**Supplementary Table 4** Continued

| **First author, year, study name** | **Vadiveloo, 2016**  **POUNDS Lost [69]** | **Yoo, 2019 NHANES III [70]** | **Zamora, 2010 CARDIA Study [71]** | **Zamora, 2011 CARDIA Study [72]** | **Zarrin, 2013**  **Nambour Skin Cancer Study [73]** |
| --- | --- | --- | --- | --- | --- |
| Appropriate, clearly focused question | Y | Y | Y | Y | Y |
| Selection from source populations comparable in all respects other than the factor under investigation | Y | Y | Y | Y | Y |
| Indication for how many of the people asked to take part did so | Y | Y | Y | Y | Y |
| Likelihood of the outcome at the time of enrolment assessed & considered | N.A. | N.A. | Y | Y | N.A. |
| Percentage of individuals that dropped out before study completion | 20.5 % | N.A.^b^ | 19 % (year 7)  28 % (year 20) | 28 % | N.A.^b^ |
| Comparison between full participants and those lost to follow-up, by exposure status | C.S. | N.A. | N | N | N.A. |
| Clearly defined outcome | Y | Y | Y | Y | Y |
| Assessment of outcome blind to exposure status | Y | N.A. | N.A. | N.A. | N.A. |
| When blinding not possible, recognition that knowledge of exposure status could have influenced assessment of outcome | Y | Y | Y | Y | Y |
| Reliable exposure assessment | C.S. | Y | Y | Y | Y |
| Evidence from other sources, that method of outcome is valid & reliable | N.A. | N.A. | N.A. | N.A. | N.A. |
| Exposure level is assessed more than once | Y | N | Y | Y | N |
| Main confounders identified and considered | N | Y | Y | Y | Y |
| Confidence intervals provided | N | Y | Y | Y | Y |
| **Quality assessment** | **+** | **++** | **+^c^** | **+^c^** | **+** |

Y: Yes; N: No; C.S.: Can`t say; N.A.: not applicable; N.A. items were excluded from the overall quality rating. +: acceptable quality; ++: high quality; ^a^21 % drop-out rate thus only acceptable quality; ^b^mortality status by date of death according to National Death Index records; ^c^28 % drop-out rate over 20 y follow-up 🡪 relatively high; missing information about: a) reasons why people dropped out, b) characteristics of drop-outs, and c) comparison between full participants and those lost to follow-up.

## Supplementary Table 5 Quality assessment of reports by the Scottish Intercollegiate Guidelines Network (SIGN) checklist which were excluded due to low quality

| **First author, year, study name** | **Chan, 2022**  **NHANES III [54]** | **Gao, 2008**  **MESA [55]** | **Hoyt, 2024, PLCO Cancer Screening Trial [57]** | **Rathod, 2012**  **NHANES III [53]** | **Thorpe, 2013**  **NHANES III [56]** |
| --- | --- | --- | --- | --- | --- |
| Appropriate, clearly focused question | Y | Y | Y | Y | Y |
| Selection from source populations comparable in all respects other than the factor under investigation | Y | C.S. | C.S. | N | C.S. |
| Indication for how many of the people asked to take part did so | N | N | N | Y | Y |
| Likelihood of the outcome at the time of enrolment is assessed and considered | N.A. | C.S. | Y | N.A. | N.A. |
| Percentage of individuals that dropped out before study completion | N.A.^a^ | 8.6 % | C.S. | N.A.^a^ | N.A.^a^ |
| Comparison between full participants and those lost to follow-up, by exposure status | N.A. | N | N | N.A. | N.A. |
| Clearly defined outcome | Y | Y | Y | Y | Y |
| Assessment of outcome blind to exposure status | N.A. | N.A. | C.S. | N.A. | N.A. |
| When blinding not possible, recognition that knowledge of exposure status could have influenced assessment of outcome | C.S. | Y | C.S. | C.S. | Y |
| Reliable exposure assessment | C.S. | Y | Y | Y | Y |
| Evidence from other sources, that method of outcome is valid and reliable | N.A. | N.A. | N | N.A. | N.A. |
| Exposure level assessed more than once | N | N | Y | N | N |
| Main confounders identified and considered | C.S. | Y | C.S. | C.S. | C.S. |
| Confidence intervals provided | Y | N | Y | Y | Y |
| **Quality assessment** | **0** | **0** | **0** | **0** | **0** |

Y: Yes; N: No; C.S.: Can`t say; N.A.: not applicable; ^a^mortality status by date of death according to National Death Index records

**Further explanations of Supplementary Table 5:**

**Chan et al., 2022:** Rejected due to questionable statistical analysis (data-driven approach to select confounders; not comparing extreme HEI categories (reference = needs improvement); very different HEI group sizes), and many uncertainties (model adjustment not entirely clear; missing descriptions and/or references in the methods section esp. regarding exposure assessment).

**Gao et al., 2008:** Rejected due to: (a) it is not clear, whether overweight/obese people at baseline are excluded from follow-up analysis, (b) missing confidence intervals (one value reported (OR or β) with a p value, (c) baseline characteristics of participants are not presented across groups of HEI.

**Hoyt et al., 2024:** Rejected due to a) characteristics of study population not presented across DQI-R categories and only for one of the two investigated study populations and b) no adjustment for socioeconomic status.

**Rathod et al., 2012:** Rejection due to insufficient information about in- and exclusion criteria, study population characteristics, potential confounding bias, and the potential of participants with baseline diseases included in analysis.

**Thorpe et al., 2013:** Rejected due to low data quality because a) weird confounding variable created as a sum of chronic conditions, b) very selective population, only non-Hispanic Black men, c) Healthy Eating Index used as a binary variable (categories: good and fair/poor), 93-98% of participants in fair/poor variable, reference group in analyses: good HEI, very different group sizes, low statistical power, and d) characteristics of participants are not presented across groups of HEI.

# References

1. The World Bank Group (2019) World Bank Country and Lending Groups. https://datahelpdesk.worldbank.org/knowledgebase/articles/906519-world-bank-country-and-lending-groups. Accessed 01 June 2019

2. World Population Review (2019) Western Countries. https://worldpopulationreview.com/country-rankings/western-countries. Accessed 01 June 2019

3. Alegria-Lertxundi, I., C. Aguirre, L. Bujanda, et al. (2020) Food groups, diet quality and colorectal cancer risk in the Basque Country. World J Gastroenterol 26(28): 4108-4125. https://doi.org/10.3748/wjg.v26.i28.4108

4. Hlaing-Hlaing, H., X. Dolja-Gore, M. Tavener, et al. (2021) Diet Quality and Incident Non-Communicable Disease in the 1946-1951 Cohort of the Australian Longitudinal Study on Women's Health. Int J Environ Res Public Health 18(21). https://doi.org/10.3390/ijerph182111375

5. Hoebeeck, L.I., E.R. Rietzschel, M. Langlois, et al. (2011) The relationship between diet and subclinical atherosclerosis: results from the Asklepios Study. Eur J Clin Nutr 65(5): 606-13. https://doi.org/10.1038/ejcn.2010.286

6. Kant, A.K. and B.I. Graubard (2005) A comparison of three dietary pattern indexes for predicting biomarkers of diet and disease. J Am Coll Nutr 24(4): 294-303. https://doi.org/10.1080/07315724.2005.10719477

7. Belin, R.J., P. Greenland, M. Allison, et al. (2011) Diet quality and the risk of cardiovascular disease: the Women's Health Initiative (WHI). Am J Clin Nutr 94(1): 49-57. https://doi.org/10.3945/ajcn.110.011221

8. Carballo-Casla, A., R. Ortolá, E. García-Esquinas, et al. (2021) The Southern European Atlantic Diet and all-cause mortality in older adults. BMC Med 19(1): 36. https://doi.org/10.1186/s12916-021-01911-y

9. Celis-Morales, C., K.M. Livingstone, A. Affleck, et al. (2018) Correlates of overall and central obesity in adults from seven European countries: findings from the Food4Me Study. Eur J Clin Nutr 72(2): 207-219. https://doi.org/10.1038/s41430-017-0004-y

10. Fallaize, R., K.M. Livingstone, C. Celis-Morales, et al. (2018) Association between Diet-Quality Scores, Adiposity, Total Cholesterol and Markers of Nutritional Status in European Adults: Findings from the Food4Me Study. Nutrients 10(1). https://doi.org/10.3390/nu10010049

11. Ford, C., S. Chang, M.Z. Vitolins, et al. (2017) Evaluation of diet pattern and weight gain in postmenopausal women enrolled in the Women's Health Initiative Observational Study. Br J Nutr 117(8): 1189-1197. https://doi.org/10.1017/s0007114517000952

12. Fung, T.T., Y. Li, S.N. Bhupathiraju, et al. (2021) Higher Global Diet Quality Score Is Inversely Associated with Risk of Type 2 Diabetes in US Women. J Nutr 151(12 Suppl 2): 168s-175s. https://doi.org/10.1093/jn/nxab195

13. Fung, T.T., S. Isanaka, F.B. Hu, et al. (2018) International food group-based diet quality and risk of coronary heart disease in men and women. Am J Clin Nutr 107(1): 120-129. https://doi.org/10.1093/ajcn/nqx015

14. Fung, T.T., Y. Li, S. Bromage, et al. (2021) Higher Global Diet Quality Score Is Associated with Less 4-Year Weight Gain in US Women. J Nutr 151(12 Suppl 2): 162s-167s. https://doi.org/10.1093/jn/nxab170

15. Fung, T.T., A. Pan, T. Hou, et al. (2016) Food quality score and the risk of coronary artery disease: a prospective analysis in 3 cohorts. Am J Clin Nutr 104(1): 65-72. https://doi.org/10.3945/ajcn.116.130393

16. Gu, X., D.D. Wang, T.T. Fung, et al. (2022) Dietary quality and risk of heart failure in men. Am J Clin Nutr 116(2): 378-385. https://doi.org/10.1093/ajcn/nqac119

17. Kohl, J., J. Brame, P. Hauff, et al. (2022) Effects of a Web-Based Weight Loss Program on the Healthy Eating Index-NVS in Adults with Overweight or Obesity and the Association with Dietary, Anthropometric and Cardiometabolic Variables: A Randomized Controlled Clinical Trial. Nutrients 15(1). https://doi.org/10.3390/nu15010007

18. Livingstone, K.M., C. Celis-Morales, S. Navas-Carretero, et al. (2016) Fat mass- and obesity-associated genotype, dietary intakes and anthropometric measures in European adults: the Food4Me study. Br J Nutr 115(3): 440-8. https://doi.org/10.1017/s0007114515004675

19. Loprinzi, P.D. and A. Nooe (2015) Health characteristics and predicted 10-year risk for a first atherosclerotic cardiovascular disease (ASCVD) event using the Pooled Cohort Risk Equations among US adults who are free of cardiovascular disease. Physiol Behav 151: 591-5. https://doi.org/10.1016/j.physbeh.2015.08.031

20. Loprinzi, P.D., E. Smit, and S. Mahoney (2014) Physical activity and dietary behavior in US adults and their combined influence on health. Mayo Clin Proc 89(2): 190-8. https://doi.org/10.1016/j.mayocp.2013.09.018

21. Mertens, E., O. Markey, J.M. Geleijnse, et al. (2018) Adherence to a healthy diet in relation to cardiovascular incidence and risk markers: evidence from the Caerphilly Prospective Study. Eur J Nutr 57(3): 1245-1258. https://doi.org/10.1007/s00394-017-1408-0

22. Mursu, J., L.M. Steffen, K.A. Meyer, et al. (2013) Diet quality indexes and mortality in postmenopausal women: the Iowa Women's Health Study. Am J Clin Nutr 98(2): 444-53. https://doi.org/10.3945/ajcn.112.055681

23. Otto, M.C., N.S. Padhye, A.G. Bertoni, et al. (2015) Everything in Moderation--Dietary Diversity and Quality, Central Obesity and Risk of Diabetes. PLoS One 10(10): e0141341. https://doi.org/10.1371/journal.pone.0141341

24. Reedy, J., E. Wirfalt, A. Flood, et al. (2010) Comparing 3 dietary pattern methods--cluster analysis, factor analysis, and index analysis--With colorectal cancer risk: The NIH-AARP Diet and Health Study. Am J Epidemiol 171(4): 479-87. https://doi.org/10.1093/aje/kwp393

25. Sharafi, M., S. Rawal, M.L. Fernandez, et al. (2018) Taste phenotype associates with cardiovascular disease risk factors via diet quality in multivariate modeling. Physiol Behav 194: 103-112. https://doi.org/10.1016/j.physbeh.2018.05.005

26. Solbak, N.M., J.Y. Xu, J.E. Vena, et al. (2017) Diet quality is associated with reduced incidence of cancer and self-reported chronic disease: Observations from Alberta's Tomorrow Project. Prev Med 101: 178-187. https://doi.org/10.1016/j.ypmed.2017.06.009

27. Tait, C.A., M.R. L'Abbé, P.M. Smith, et al. (2020) Adherence to Predefined Dietary Patterns and Risk of Developing Type 2 Diabetes in the Canadian Adult Population. Can J Diabetes 44(2): 175-183.e2. https://doi.org/10.1016/j.jcjd.2019.06.002

28. Arthur, R.S., V.A. Kirsh, and T.E. Rohan (2023) The association of the healthy eating index with risk of colorectal cancers (overall and by subsite) among Canadians. Cancer Epidemiol 87: 102454. https://doi.org/10.1016/j.canep.2023.102454

29. Das, S.K., R.E. Silver, A. Senior, et al. (2023) Diet composition, adherence to calorie restriction, and cardiometabolic disease risk modification. Aging Cell 22(12): e14018. https://doi.org/10.1111/acel.14018

30. Hill, E.B., L.T. Cubellis, R.K. Wexler, et al. (2023) Differences in Adherence to American Heart Association's Life's Essential 8, Diet Quality, and Weight Loss Strategies Between Those With and Without Recent Clinically Significant Weight Loss in a Nationally Representative Sample of US Adults. J Am Heart Assoc 12(8): e026777. https://doi.org/10.1161/jaha.122.026777

31. Hill, E.B., I.R. Konigsberg, D. Ir, et al. (2023) The Microbiome, Epigenome, and Diet in Adults with Obesity during Behavioral Weight Loss. Nutrients 15(16). https://doi.org/10.3390/nu15163588

32. Kim, J., Y. Zhang, H. Kim, et al. (2023) A Comparative Study of Healthy Dietary Patterns for Incident and Fatal Digestive System Cancer. Am J Gastroenterol 118(11): 2061-2070. https://doi.org/10.14309/ajg.0000000000002448

33. Li, K., Y. Huang, L. Wang, et al. (2024) Association of Four Dietary Patterns and Stair Climbing with Major Adverse Cardiovascular Events: A Large Population-Based Prospective Cohort Study. Nutrients 16(21). https://doi.org/10.3390/nu16213576

34. Maitland, S.B., P. Brauer, D.M. Mutch, et al. (2024) Exploratory analysis of the variable response to an intensive lifestyle change program for metabolic syndrome. BMC Prim Care 25(1): 357. https://doi.org/10.1186/s12875-024-02608-w

35. Shang, X., J. Liu, Z. Zhu, et al. (2023) Healthy dietary patterns and the risk of individual chronic diseases in community-dwelling adults. Nat Commun 14(1): 6704. https://doi.org/10.1038/s41467-023-42523-9

36. Wang, Y.B., A.J. Page, T.K. Gill, et al. (2023) The association between diet quality, plant-based diets, systemic inflammation, and mortality risk: findings from NHANES. Eur J Nutr 62(7): 2723-2737. https://doi.org/10.1007/s00394-023-03191-z

37. Yao, H., X. Wang, X. Wu, et al. (2024) Sex differences in association of healthy eating pattern with all-cause mortality and cardiovascular mortality. BMC Public Health 24(1): 2363. https://doi.org/10.1186/s12889-024-19883-y

38. Yuan, S., J. He, S. Wu, et al. (2023) Trends in dietary patterns over the last decade and their association with long-term mortality in general US populations with undiagnosed and diagnosed diabetes. Nutr Diabetes 13(1): 5. https://doi.org/10.1038/s41387-023-00232-8

39. Zhang, Y., K.H. Stopsack, M. Song, et al. (2024) Healthy dietary patterns and risk of prostate cancer in men at high genetic risk. Int J Cancer 155(1): 71-80. https://doi.org/10.1002/ijc.34898

40. Gopinath, B., E. Rochtchina, V.M. Flood, et al. (2013) Diet quality is prospectively associated with incident impaired fasting glucose in older adults. Diabet Med 30(5): 557-62. https://doi.org/10.1111/dme.12109

41. Lv, Y., V.B. Kraus, X. Gao, et al. (2020) Higher dietary diversity scores and protein-rich food consumption were associated with lower risk of all-cause mortality in the oldest old. Clin Nutr 39(7): 2246-2254. https://doi.org/10.1016/j.clnu.2019.10.012

42. McCrory, M.A., P.J. Fuss, J.E. McCallum, et al. (1999) Dietary variety within food groups: association with energy intake and body fatness in men and women. Am J Clin Nutr 69(3): 440-7. https://doi.org/10.1093/ajcn/69.3.440

43. Torres-Collado, L., M. García-de la Hera, N. Cano-Ibañez, et al. (2022) Association between Dietary Diversity and All-Cause Mortality: A Multivariable Model in a Mediterranean Population with 18 Years of Follow-Up. Nutrients 14(8). https://doi.org/10.3390/nu14081583

44. Embling, R., M.J. Price, M.D. Lee, et al. (2023) Associations between dietary variety, portion size and body weight: prospective evidence from UK Biobank participants. Br J Nutr 130(7): 1267-1277. https://doi.org/10.1017/s0007114523000156

45. Zheng, G., M. Cai, H. Liu, et al. (2023) Dietary Diversity and Inflammatory Diet Associated with All-Cause Mortality and Incidence and Mortality of Type 2 Diabetes: Two Prospective Cohort Studies. Nutrients 15(9). https://doi.org/10.3390/nu15092120

46. Mozaffari, H., F. Imamura, R.A. Murphy, et al. (2025) Protein diversity, type 2 diabetes, and effect modifiers: a multi-country prospective study. Int J Epidemiol 54(3). https://doi.org/10.1093/ije/dyaf057

47. Wiese, M.L., F. Frost, M. Bahls, et al. (2025) Dietary Diversity, Rather Than Quality, Parallels a Reduction in Metabolic Syndrome and a Favorable Gut Microbiome: The Dietary Diversity Score. J Am Nutr Assoc 44(3): 256-266. https://doi.org/10.1080/27697061.2024.2423775

48. Jessri, M., D. Hennessey, A. Bader Eddeen, et al. (2023) Mortality and Life Expectancy Lost in Canada Attributable to Dietary Patterns: Evidence From Canadian National Nutrition Survey Linked to Routinely Collected Health Administrative Databases. Am J Epidemiol 192(3): 377-396. https://doi.org/10.1093/aje/kwac189

49. Bray, G.A., L. Qi, and F.M. Sacks (2024) Is There an Ideal Diet? Some Insights from the POUNDS Lost Study. Nutrients 16(14). https://doi.org/10.3390/nu16142358

50. Liu, D., X.R. Zhang, Z.H. Li, et al. (2021) Association of dietary diversity changes and mortality among older people: A prospective cohort study. Clin Nutr 40(5): 2620-2629. https://doi.org/10.1016/j.clnu.2021.04.012

51. Park, S. (2024) Association of a High Healthy Eating Index Diet with Long-Term Visceral Fat Loss in a Large Longitudinal Study. Nutrients 16(4). https://doi.org/10.3390/nu16040534

52. Heidari, S., M. Kahnooji, F. Ayoobi, et al. (2025) Relationship between metabolic syndrome and dietary diversity in the Rafsanjan cohort study. Scientific Reports 15(1): 10579. https://doi.org/10.1038/s41598-025-90086-0

53. Rathod, A.D., A.S. Bharadwaj, A.O. Badheka, et al. (2012) Healthy Eating Index and mortality in a nationally representative elderly cohort. Arch Intern Med 172(3): 275-7. https://doi.org/10.1001/archinternmed.2011.1031

54. Chan, J.E., M.A. Caesar, A.K. Mann, et al. (2022) The Role of Diet Compared to Physical Activity on Women's Cancer Mortality: Results From the Third National Health and Nutrition Examination Survey. Front Public Health 10: 853636. https://doi.org/10.3389/fpubh.2022.853636

55. Gao, S.K., S.A. Beresford, L.L. Frank, et al. (2008) Modifications to the Healthy Eating Index and its ability to predict obesity: the Multi-Ethnic Study of Atherosclerosis. Am J Clin Nutr 88(1): 64-9. https://doi.org/10.1093/ajcn/88.1.64

56. Thorpe, R.J., Jr., S.M. Wilson-Frederick, J.V. Bowie, et al. (2013) Health behaviors and all-cause mortality in African American men. Am J Mens Health 7(4 Suppl): 8s-18s. https://doi.org/10.1177/1557988313487552

57. Hoyt, M., Y. Song, S. Gao, et al. (2024) Associations between Two Dietary Quality Scores and Pancreatic Cancer Risk in a US National Prospective Cohort Study. J Am Nutr Assoc 43(4): 345-355. https://doi.org/10.1080/27697061.2023.2289520

58. Alhazmi, A., E. Stojanovski, M. McEvoy, et al. (2014) Diet quality score is a predictor of type 2 diabetes risk in women: the Australian Longitudinal Study on Women's Health. Br J Nutr 112(6): 945-51. https://doi.org/10.1017/s0007114514001688

59. Arabshahi, S., J.C. van der Pols, G.M. Williams, et al. (2012) Diet quality and change in anthropometric measures: 15-year longitudinal study in Australian adults. Br J Nutr 107(9): 1376-85. https://doi.org/10.1017/s0007114511004351

60. Fung, T.T., F.B. Hu, M.L. McCullough, et al. (2006) Diet quality is associated with the risk of estrogen receptor-negative breast cancer in postmenopausal women. J Nutr 136(2): 466-72. https://doi.org/10.1093/jn/136.2.466

61. Harnack, L., K. Nicodemus, D.R. Jacobs, Jr., et al. (2002) An evaluation of the Dietary Guidelines for Americans in relation to cancer occurrence. Am J Clin Nutr 76(4): 889-96. https://doi.org/10.1093/ajcn/76.4.889

62. Kappeler, R., M. Eichholzer, and S. Rohrmann (2013) Meat consumption and diet quality and mortality in NHANES III. Eur J Clin Nutr 67(6): 598-606. https://doi.org/10.1038/ejcn.2013.59

63. Lassale, C., L. Fezeu, V.A. Andreeva, et al. (2012) Association between dietary scores and 13-year weight change and obesity risk in a French prospective cohort. Int J Obes (Lond) 36(11): 1455-62. https://doi.org/10.1038/ijo.2011.264

64. McCullough, M.L., D. Feskanich, M.J. Stampfer, et al. (2000) Adherence to the Dietary Guidelines for Americans and risk of major chronic disease in women. Am J Clin Nutr 72(5): 1214-22. https://doi.org/10.1093/ajcn/72.5.1214

65. McCullough, M.L., D. Feskanich, E.B. Rimm, et al. (2000) Adherence to the Dietary Guidelines for Americans and risk of major chronic disease in men. Am J Clin Nutr 72(5): 1223-31. https://doi.org/10.1093/ajcn/72.5.1223

66. Nickel, D.V., F. Jannasch, E. Inan-Eroglu, et al. (2024) Healthy food diversity and the risk of major chronic diseases in the EPIC-Potsdam study. Sci Rep 14(1): 28635. https://doi.org/10.1038/s41598-024-78287-5

67. Park, Y.M., T.T. Fung, S.E. Steck, et al. (2016) Diet Quality and Mortality Risk in Metabolically Obese Normal-Weight Adults. Mayo Clin Proc 91(10): 1372-1383. https://doi.org/10.1016/j.mayocp.2016.06.022

68. Shahar, D.R., B. Yu, D.K. Houston, et al. (2009) Dietary factors in relation to daily activity energy expenditure and mortality among older adults. J Nutr Health Aging 13(5): 414-20. https://doi.org/10.1007/s12603-009-0077-y

69. Vadiveloo, M., F.M. Sacks, C.M. Champagne, et al. (2016) Greater Healthful Dietary Variety Is Associated with Greater 2-Year Changes in Weight and Adiposity in the Preventing Overweight Using Novel Dietary Strategies (POUNDS Lost) Trial. J Nutr 146(8): 1552-9. https://doi.org/10.3945/jn.115.224683

70. Yoo, E.R., D. Kim, L.M. Vazquez-Montesino, et al. (2020) Diet quality and its association with nonalcoholic fatty liver disease and all-cause and cause-specific mortality. Liver Int 40(4): 815-824. https://doi.org/10.1111/liv.14374

71. Zamora, D., P. Gordon-Larsen, D.R. Jacobs, Jr., et al. (2010) Diet quality and weight gain among black and white young adults: the Coronary Artery Risk Development in Young Adults (CARDIA) Study (1985-2005). Am J Clin Nutr 92(4): 784-93. https://doi.org/10.3945/ajcn.2010.29161

72. Zamora, D., P. Gordon-Larsen, K. He, et al. (2011) Are the 2005 Dietary Guidelines for Americans Associated With reduced risk of type 2 diabetes and cardiometabolic risk factors? Twenty-year findings from the CARDIA study. Diabetes Care 34(5): 1183-5. https://doi.org/10.2337/dc10-2041

73. Zarrin, R., T.I. Ibiebele, and G.C. Marks (2013) Development and validity assessment of a diet quality index for Australians. Asia Pac J Clin Nutr 22(2): 177-87. https://doi.org/10.6133/apjcn.2013.22.2.15
